# Effect of online infographics for enhancing health literacy among patients with type 2 diabetes in primary care unit during the COVID-19 pandemic: a randomized controlled trial

**DOI:** 10.1186/s12875-024-02335-2

**Published:** 2024-03-15

**Authors:** Suchada Sutthiworapon, Polathep Vichitkunakorn, Kittisakdi Choomalee, Pitchayanont Ngamchaliew

**Affiliations:** Department of Family and Preventive Medicine, Faculty of Medicine, Prince of Songkla University, Hat Yai, Songkhla, 90110 Thailand

**Keywords:** Health literacy, Knowledge, Infographic, Media intervention, Type 2 diabetes mellitus, Health education, Primary care

## Abstract

**Background:**

Health literacy (HL) in patients with type 2 diabetes mellitus (DM) can help control disease and prevent complications. However, most patients with type 2 DM have inadequate HL; therefore, their HL must be further improved. This study aimed to determine the effects of online infographics on improving HL among patients with type 2 DM.

**Methods:**

This randomized controlled trial was conducted from July 2022 to September 2022, at the primary care unit of Songklanagarind Hospital, Thailand; 30 patients with type 2 DM were randomly assigned to the experimental (*n* = 15; three types of infographics) and control (*n* = 15; three types of pamphlets) groups. Infographics and pamphlets were distributed weekly via social media platforms. The S-TOFHLA Thai version and Thai-FCCHL were used to evaluate HL. Chi-square, Fisher’s exact, Wilcoxon rank-sum, t-test, paired t-test, and McNemar’s chi-square tests were used.

**Results:**

The median age of 30 participants was 56 years. The mean duration of DM was 9.6 years, with a median HbA1c level of 7.5 mg%. Most participants (80%) had adequate HL in S-TOFHLA, whereas 63.3% had adequate HL in FCCHL. All participants in the infographic group who had inadequate HL in the S-TOFHLA pre-test achieved adequate HL. Meanwhile, only 50% of patients in the pamphlet group achieved adequate HL. Regarding FCCHL, 50% of patients in the infographic group and 60% in the pamphlet group who had inadequate HL in the pretest achieved adequate HL. However, no statistical significance in achieving adequate HL was found in either group. The mean differences (SD) in S-TOFHLA between before and after intervention were 12.53 (8.77; *p* = 0.0007) and 10.13 (9.88; *p* = 0.001) in the infographic and pamphlet groups, respectively. Regarding FCCHL, the mean differences (SD) were 3.47 (4.29) and 3.20 (2.91) in the infographic group (*p* = 0.003) and pamphlet (*p* = 0.002) groups, respectively. No statistical significance in the mean difference was found between both groups.

**Conclusions:**

Novel online infographics and pamphlets did not significantly differ in achieving adequate HL among patients with type 2 DM who should receive health education about disease control and complication prevention. However, both interventions can increase and maintain HL levels. Online educational media can be appropriate during the COVID-19 pandemic. Nevertheless, further larger-scale studies should be performed to examine the impact of other DM educational media on HL promotion.

**Trial registration:**

The Thai Clinical Trials Registry (TCTR) with registry ID TCTR20230425001 (date of registration 25/04/2023).

**Supplementary Information:**

The online version contains supplementary material available at 10.1186/s12875-024-02335-2.

## Background

Type 2 diabetes mellitus (DM) is a noncommunicable disease (NCD) and a major health problem globally, including in Thailand. Type 2 DM accounts for 90%–95% of all diabetes cases [1]. It results in micro- and macrovascular complications that can cause chronic diseases, disabilities, and even death. The International Diabetes Federation reported that 425 million people worldwide had DM in 2017 and that 629 million individuals will have DM in 2045 [2]. The Thai National Health Examination Survey VI reported that the prevalence of DM increased from 8.9% in 2014 to 9.5% in 2020. The prevalence rises with age and is highest in those aged 60–69 years [3].

Uncontrolled type 2 DM will lead to complications. Agrawal et al. revealed that vascular complications of type 2 DM include diabetes retinopathy (32.5%), diabetes nephropathy (30.2%), peripheral vascular diseases (28%), diabetes neuropathy (26.8%), and cardiovascular diseases (25.8%). These complications affect patients’ quality of life, families, society, and country [4]. DM complications are influenced by several risk factors, including self-care behaviors such as diet control, exercise, and medications to reduce blood sugar levels and prevent DM complications. DM management can influence the quality of life of individuals. Medication adherence, dietary restrictions, and lifestyle modifications may affect the well-being of individuals with DM [5]. DM is incurable. Therefore, maintaining blood glucose levels at an appropriate range requires both self-care and patient cooperation. Health literacy (HL) is the one of the main factors that might contribute to obtain optimal self-care behaviors [6].

HL refers to the ability and skills to access information, knowledge, and understanding to evaluate self-care management and provide people, families, and communities with health guidance for good health [7]. HL emphasizes on a person’s competencies and skills, based on six basic characteristics, namely, access to health information, cognition, communication skills, decision-making skills, media literacy, and self-management [7]. Because HL is associated with health outcomes, patients with DM who have adequate HL and appropriate healthcare behaviors can effectively control their blood sugar levels, leading to the prevention of complications. Tefera YG et al. reported that patients with a high HL are 1.85 times more likely to achieve the target glycemic control than those with a low HL (AOR: 1.85 [1.09–3.40]) [8]. Schillinger et al. indicated that patients with adequate HL had 2.03 times better glycemic control than those with inadequate HL. Additionally, they found that patients with inadequate HL had a 2.33 times higher risk of developing DM renal complications and 2.71 times more likely to have a stroke than patients with adequate HL [9]. Moreover, Saeed et al. revealed that inadequate HL can be associated with poor glycemic control (HbA1C > 9%) and DM complications, particularly retinopathy (OR = 13.1, *p* = 0.003) [10]. Breder et al. reported that individuals with inadequate HL (32.8%) had a higher risk of diabetic retinopathy than those with adequate HL (16.5%) (*P* = 0.0081) [11]. Hadden K showed that the diabetic foot amputation group was 8.07 times more likely to have inadequate HL than the general orthopedic patient group [12]. However, HL can be improved by various techniques, such as the fotonovela technique (using images), teach-back technique, shame-free technique, checklists, and scorecards [7]. Previous studeies have indicated that group-based education is frequently used to enhance HL in people with DM, and it has been successful in promoting HL in patients with DM [13, 14]. Therefore, educating individuals diagnosed with type 2 DM regarding specific techniques can enhance their HL.

Group teaching was limited during the COVID-19 pandemic. Therefore, novel online teaching techniques were used to enhance HL. Infographics, which are picture-based educational media, may be more appropriate for online distribution. HL was provided via infographics, which comprised information and graphics. People commonly learn a story better from visualization than from the text-based approach. Infographics are easy to understand because of their visually appealing, brief, and concise functions. They are often shared on social media, thereby making them easily accessible to a large group of people. Furthermore, they are visually appealing, and infographic-based education is appropriate for the public who values convenience and has a rapid lifestyle [15]. The LINE application is a widely used communication application and platform in Thailand. The application offers various communication features, including text messaging, voice and video calls, and multimedia content sharing. LINE is available on various devices such as smartphones, tablets, and computers [16].

Adequate HL in type 2 DM will lead to lifestyle modifications, self-care behaviors, and medical adherence to control blood glucose levels and reduce the development of DM complications [8, 10–12]. Therefore, this study aimed to compare the effects of infographics and conventional health education (i.e., provision of DM information pamphlets to patients with DM who visited for healthcare services) distributed on LINE application on improving HL among patients with type 2 DM at the primary care unit (PCU) of a tertiary hospital in southern Thailand.

## Methods

### Study design and setting

A randomized controlled trial study was conducted among patients with type 2 DM, who visited the PCU of Songklanagrarind Hospital between July 1, 2022, and September 30, 2022.

### Population and sample

This study included patients aged 35–60 years who presented with type 2 DM determined using the ICD-10 codes E11–E11.9, lived in Hat Yai, Songkhla, could communicate and understand written Thai, could access or had a family member that could access the Internet via a mobile device (i.e., smartphone or tablet), and had hemoglobin A1C (HbA1C) values recorded in the hospital information system within the last 3 months. Participants who refused to answer the survey, who refused to follow-up for the final evaluation at home, or from whom the questionnaire could not be obtained were excluded.

There were 890 patients with type 2 DM in the PCU of Songklanagarind Hospital in 2021. The sample size used in the randomized controlled trial was based on a clinical trial comparing continuous outcomes between groups in the RCT or cluster RCT formula with the n4Studies software, which was calculated based on the study by Kim et al. [17]. The mean (standard deviation [SD]) of HL score in the treatment and control groups were 58.25 (4.7) and 51.76 (6.15), respectively, with a margin of error of 0.05. Thus, each group should have 12 participants. This study added 20% more participants to prevent data errors; as a result, 15 participants were considered for both the experimental and control groups.

### Study instrument

In this study, data were gathered using self-administered questionnaires, which included the following parts: the sociodemographic characteristic questionnaires included age, sex, education (primary school or below/secondary school or higher), employment status (employed/unemployed), marital status (married, widowed, single, or separated), monthly income, DM duration, and comorbidities (i.e., hypertension, dyslipidemia, and cerebrovascular diseases). This study measured the levels of HL using two standard instruments that were translated into Thai, i.e., the Thai version of the Short Test of Functional Health Literacy in Adults (S-TOFHLA Thai version) and the Thai version of Functional, Communicative, Critical Health Literacy (Thai-FCCHL).

### S-TOFHLA Thai version

The S-TOFHLA Thai version was developed using the original “Short test of functional health literacy in adults” by David Baker et al. [18], wherein the HL of adults was measured by assessing both numeracy and reading comprehension using actual health-related materials. The questionnaire consisted of the following five dimensions: reading skills, information accessibility, information comprehension, information analysis and decision making, and information behavior. The sum of in five dimensions of the S-TOFHLA score ranges from 0 to 100. S-TOFHLA scores are classified into three levels, namely, low HL (0–53 points), medium HL (54–66 points), and high HL (67–100 points). The validity and reliability of the questionnaire have been confirmed in previous studies and were tested using a Cronbach’s alpha value of 0.916 [19]. The correlation coefficient between S-TOFHLA and REALM (0.80) was slightly lower than that between TOFHLA and REALM (0.84) in the original development study [20]. This study divided the S-TOFHLA score levels into inadequate and adequate HL. High levels of HL were categorized as an adequate HL group, whereas low and medium levels were categorized as an inadequate HL group.

### Thai-FCCHL

The Thai-FCCHL was developed using the original “Functional, Communicative and Critical Health Literacy Scale (FCCHL),” by Ishikawa Hirono et al. [21], which was used to assess the HL of patients with DM. The questionnaire consisted of the following three dimensions: functional HL, communicative HL, and critical HL. The sum of the three dimensions of the FCCHL score, which ranges from 14 to 56, was classified into low HL (14–28 points), medium HL (29–42 points), and high HL (43–56 points). The validity and reliability of the questionnaire have been confirmed in previous studies and were tested using a content validity index (CVI) of 0.78 and a Cronbach’s alpha value of 0.91 [22, 23]. This study divided the FCCHL score levels into inadequate and adequate HL. High levels of HL were categorized as adequate HL, whereas low and medium levels of HL were categorized as inadequate HL.

### Data collection process

**Fig. 1:**
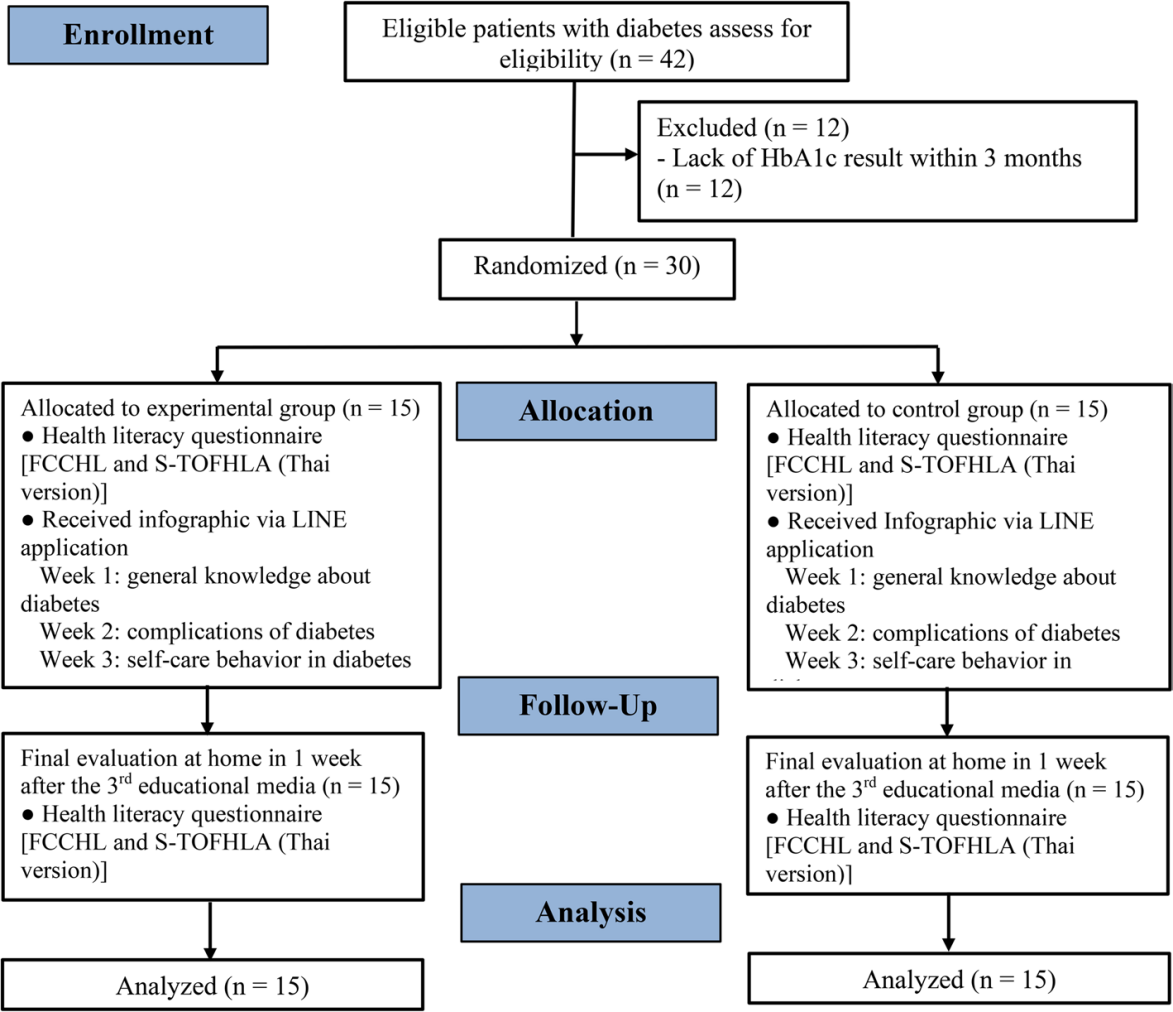
Study flow

Figure 1 shows the data collection process. During the initial visit, a primary care officer at the PCU of Songklanagrarind Hospital informed the patients with type 2 DM about the study during their visits. Patients who were interested in participating in the study completed a screening questionnaire to determine their eligibility. Patients who were eligible were provided with a letter of information and were instructed to sign the consent form. Eligible participants who visited the PCU were randomly assigned to the experimental and control groups via simple random sampling. Allocation concealment was implemented using the opaque, sealed envelope technique, which was opened by the author. The participants were not blinded to the allocation because of the nature of the intervention. After the initial coordination and allocation of participants to either the experimental or control group, the authors instructed them to complete the initial questionnaire at the PCU. The first data points collected included the sociodemographic and HL questionnaires, including the Thai versions of S-TOFHLA and FCCHL.

Each group received intervention. The experimental group received DM education with an “infographic” containing information. In this intervention, a set of three picture-based educational materials, with some keyword texts within three weekly sessions, was provided. The control group received the usual DM education using a “pamphlet” containing information. In this intervention, three text-based educational materials, with a few added illustrations, were provided within three weekly sessions. Both educational sessions were also administered to the participants via the LINE application. To validate whether the participants received and read the infographics or pamphlets that were sent via the LINE application, all participants were contacted via telephone to confirm the type of educational media they had obtained. The authors conducted weekly telephone calls to validate whether the participants in both groups had received and read the educational media. The telephone calls did not provide any further information or knowledge.

Educational media based on the Standard of Medical Care in Diabetes, American Diabetes Association (ADA) 2017, for was given for each group, and educational content and intervention procedures were developed [5]. The educational content of infographics and pamphlets was consistent. Both infographics and pamphlets were tested for their content validity by three experts, including a family physician, internist, and health academician, who assessed and confirmed the content validity index (CVI). The CVI values in infographics and pamphlets were 0.83 and 0.81, respectively. The type 2 DM education media content is presented in Supplementary 1, 2 and 3.

After giving both groups with educational media about DM, within three weekly sessions, the two groups underwent the final evaluation conducted by the author at the patients’ homes at 1 week after the last distribution of the third educational media. HL questionnaire used for collecting post-experimental data included the Thai versions of S-TOFHLA and FCCHL. The study flowchart is presented in Fig. 1.

Experimental data were gathered and then used for the data analysis. Each patient was assigned an ID number; however, personally identifiable information was not stored, and individual confidentiality was maintained.

### Data editing

To detect any inaccuracy in the records, data were entered twice in a computer database management system by Microsoft Excel, version 2019.

### Statistical analysis

R software version 4.1.1 was used to analyze data. Sociodemographic data (i.e., age, sex, education, employment status, marital status, monthly income, DM duration, and comorbidities) were analyzed by descriptive statistics [percentage, interquartile (IQR), mean and standard deviation (SD)]. We performed the Chi-square test and Fisher's exact test on categorical variables to compare the intervention and control groups. For continuous variables, we used the Wilcoxon rank-sum test and the t-test. To compare the scores and the proportion of change from inadequate HL to adequate HL between the pre-test and post-test groups, we utilized McNemar's Chi-square test for paired proportions and the paired t-test for continuous outcomes. We verified data normality using histograms for visual inspection and the Shapiro–Wilk test, which confirmed that the data had a normal distribution (*p* > 0.05). A *p*-value of < 0.05 was considered statistically significant.

## Results

### Baseline characteristics

The study enrolled 30 participants, who were then divided into either the infographic group or the pamphlet group, with 15 participants in each. There was no dropout rate for follow-up in this study.

Table 1 shows the socioeconomic characteristics and clinical baseline of the participants, 83.3% were female, 56.7% had an educational level below primary school, 56.7% were married, and 60% were employed. The median (interquartile ratio [IQR]) monthly income was 15,000 (4,750, 20,000) THB. The most common comorbidities were hypertension (75.9%) and dyslipidemia (72.4%). The median (IQR) HbA1C was 7.5 (7, 7.9) mg%. Most participants had a mean (standard deviation [SD]) of DM duration was 9.6 (7.5) years; however, the infographic group had a significantly lower mean (SD) of DM duration than the pamphlet group, with 6.6 (5.3) and 12.3 (8.3) years, respectively (*p* = 0.036). Moreover, the infographic group had significantly lower median (IQR) of age than the pamphlet group, i.e., 55 (47, 56) and 58 (54, 60) years, respectively (*p* = 0.042).

**Table 1:** Baseline sociodemographic characteristics, clinical data, and health literacy levels between the infographic and pamphlet groups (*n* = 30)

Variable	Total (*n* = 30)	Infographic group (*n* = 15)	Pamphlet group (*n* = 15)	*p*-value
**Age: median (IQR)**	56 (50.2, 58.8)	55 (47, 56)	58 (54, 60)	0.042^R*^
**Sex [n (%)]**				
Male	5 (16.7)	3 (20)	2 (13.3)	1^F^
Female	25 (83.3)	12 (80)	13 (86.7)	
**Education [n (%)]**				
Primary school or below	17 (56.7)	9 (60)	8 (53.3)	0.713^C^
Secondary school or higher	13 (43.3)	6 (40)	7 (46.7)	
**Marital status [n (%)]**				
Married	17 (56.7)	9 (60)	8 (53.3)	0.713^C^
Widowed/single/separated	13 (43.3)	6 (40)	7 (46.7)	
**Occupational [n (%)]**				
Employed	18 (60)	10 (66.7)	8 (53.3)	0.456^C^
Unemployed	12 (40)	5 (33.3)	7 (46.7)	
**Monthly income** **(Thai baht) Median (IQR)**	15,000 (4,750, 20,000)	17,500 (13,750, 20,000)	10,000 (3,750, 17,000)	0.143^R^
**Comorbidities [n (%)]**				
Hypertension	22 (75.9)	11 (78.6)	11 (73.3)	1^F^
Dyslipidemia	21 (72.4)	11 (78.6)	10 (66.7)	0.682^F^
Cerebrovascular disease	1 (3.3)	1 (7.1)	0 (0)	0.483^F^
**Diabetes duration** **(years, mean [SD])**	9.6 (7.5)	6.6 (5.3)	12.3 (8.3)	0.036^T*^
**HbA**_**1**_**C** **[mg%, median (IQR)]**	7.5 (7, 7.9)	7.5 (7, 7.8)	7.1 (7, 8)	0.662^R^
**S-TOFHLA Thai version: pre-test**				
Mean (SD)	77.30 (11.64)	75.67 (11.84)	78.93 (11.61)	0.942^ T^
**Inadequate (0–66) [n (%)]**	6 (20)	4 (26.7)	2 (13.3)	0.651^F^
Low (0–53)	2 (6.7)	1 (6.7)	1 (6.7)	
Medium (54–66)	4 (13.3)	3 (20)	1 (6.7)	
**Adequate (High = 67–100) [n (%)]**	24 (80)	11 (73.3)	13 (86.7)	
**FCCHL Thai version: pre-test**				
Mean (SD)	42.77 (4.43)	41.47 (5,67)	44.07 (2.22)	0.115^ T^
**Inadequate (14–42) [n (%)]**	11 (36.7)	6 (40)	5 (33.3)	
Low (14–28)	1 (3.3)	1 (6.7)	0 (0)	0.705^C^
Medium (29–42)	10 (33.3)	5 (33.3)	5 (33.3)	
**Adequate (High: 43–56) [n (%)]**	19 (63.3)	9 (60)	10 (66.7)	

^F^Fisher exact test

^R^Rank-sum test

^C^Chi-square test

^T^T-test

^*^p < 0.05

As shown in Table 1, the mean baseline S-TOFHLA scores were 75.67 (SD: 11.84) in the infographic group and 78.93 (SD: 11.61) in the pamphlet group (*p* = 0.942). If classified according to HL levels based on S-TOFHLA, the participants (80%) commonly had adequate HL. That is, the HLs were 73.3% in the infographic group and 86.7% in the pamphlet group (*p* = 0.651). The mean baseline FCCHL scores were 41.47 (SD: 5.67) in the infographic group and 44.07 (SD: 2.22) in the pamphlet group (*p* = 0.115). When classified into levels of HL by the FCCHL, most participants (63.3%) had adequate HL, with 60% in the infographic group and 66.7% in the pamphlet group (*p* = 0.705). No statistically significant difference in the baseline score and level of HL was found between the two groups.

### HL changes after the intervention between the infographic and pamphlet groups

As shown in Table 2, all participants in the infographic group, who had inadequate HL in the pre-test of S-TOFHLA, could reach adequate HL, whereas half of the participants in the pamphlet group, who had inadequate HL in the pre-test, could reach adequate HL. However, no statistically significant difference in attaining higher HL was found between the two groups (*p* = 0.1336 and *p* = 1, respectively).

**Table 2:** Percentage of changes to participants with adequate health literacy after the intervention between the infographic and pamphlet groups (*n* = 30)

Health literacy	Group	Pretest n (%)		Post-test		*p*-value
				**Inadequate** **n (row %)**	**Adequate** **n (row %)**	
**S-TOFHLA** **Thai version**	Infographic group (*n* = 15)	Inadequate HL	4 (26.7)	0 (0)	4 (100)	0.1336^M^
		Adequate HL	11 (73.3)	0 (0)	11 (100)	
	Pamphlet group (*n* = 15)	Inadequate HL	2 (13.3)	1 (50)	1 (50)	1^M^
		Adequate HL	13 (86.7)	0 (0)	13 (100)	
**FCCHL** **Thai version**	Infographic group (*n* = 15)	Inadequate HL	6 (40)	3 (50)	3 (50)	0.2482^M^
		Adequate HL	9 (60)	0 (0)	9 (100)	
	Pamphlet group (*n* = 15)	Inadequate HL	5 (33.3)	2 (40)	3 (60)	0.2482^M^
		Adequate HL	10 (66.7)	0 (0)	10 (100)	

^M^McNemar’s Chi-square test

Regarding the FCCHL, half of the participants in the infographic group, who had inadequate HL in the pretest, could achieve adequate HL, whereas 60% of participants in the pamphlet group, who had inadequate HL in the pre-test, could achieve adequate HL. However, no statistically significant difference in HL upgrade was found between the two groups (*p* = 0.2482 and *p* = 0.2482, respectively).

As presented in Table 3, the mean (SD) of the post-test S-TOFHLA in the infographic group was 88.20 (6.14). The mean difference, when compared with the pretest, was 12.53 (8.77). A statistically significant difference in the mean scores was found (*p* = 0.0007). The mean (SD) of the post-test S-TOFHLA in the pamphlet group was 89.07 (8.37). The mean difference, when compared with the pretest was 10.13 (9.88), and a statistically significant difference in mean scores was observed (*p* = 0.001). However, when comparing the mean difference scores between groups, no statistically significant difference was found in the mean scores between the groups (*p* = 0.115).

**Table 3:** Mean difference in the health literacy score between the infographic group and the pamphlet group (*n* = 30)

Health literacy	Pretest		Post-test		Mean difference	SD	*p*-value (pre-test and post-test within the group)	*p*-value (mean difference between groups)
	**Mean**	**SD**	**Mean**	**SD**				
**S-TOFHLA Thai version**								
Infographic group	75.67	11.84	88.20	6.14	12.53	8.77	< 0.001^P*^	0.115^T^
Pamphlet group	78.93	11.61	89.07	8.37	10.13	9.88	0.001^P*^	
**FCCHL Thai version**								
Infographic group	41.47	5.67	44.93	3.26	3.47	4.29	0.003^P*^	0.942^T^
Pamphlet group	44.07	2.22	47.27	2.94	3.20	2.91	0.002^P*^	

^P^Paired t-test

^T^T-test

^*^p < 0.05

Regarding the FCCHL used to measure HL, the mean (SD) of the post-test in the infographic group was 44.93 (3.26), and the mean difference when compared with the pre-test was 3.47 (4.29). A statistically significant difference in mean scores was noted (*p* = 0.003). The mean (SD) of the post-test FCCHL score in the pamphlet group was 47.27 (2.94). The mean difference, when compared with the pretest, was 3.20 (2.91), and a statistically significant difference was found in the mean scores (*p* = 0.002). However, when comparing the mean difference in scores between the infographic group and the pamphlet group, no statistically significant difference in the mean difference scores was found between the groups (*p* = 0.942).

## Discussion

Previous studies have shown that HL is associated with clinical health outcomes, and promoting HL will result in its adequacy and appropriate health behaviors, leading to the effective control of blood sugar levels and reduction or slowing down of the occurrence of complications [9]. This main study outcome classified the S-TOFHLA and FCCHL scores into two categories (inadequate and adequate HL levels) to determine the HL level of the study participants. This study showed that nearly all participants who had inadequate HL in the S-TOFHLA and FCCHL pretests achieved adequate HL. However, no statistically significant difference was found in reaching adequate HL between the two groups. This might be explained by the fact that the patients in both groups had adequate or high baseline HL levels. As participants with adequate or high HL levels were not excluded from the study, the results might not reflect real-world study outcomes. Therefore, this could have caused selection bias. This study might yield internal validity as the baseline sociodemographic characteristics of the participants, including age and duration of type 2 DM, which slightly differed between the two groups, were heterogeneous. The heterogenicity of both groups, particularly in terms of DM duration, may have affected the HL level of the participants. This could be explained by the fact that patients with long-term type 2 DM typically have a better HL and self-care DM management than those with short-term type 2 DM [24–26]. Therefore, a longer type 2 DM duration in the pamphlet group might indicate an internal validity that influenced the study findings. Moreover, health education is a standard recommendation for all patients with DM, without discriminating between patients in the inadequate or adequate HL group, according to the Standard of Medical Care in Diabetes, ADA (2017) [5]. The study participants had high or adequate HL levels at baseline; therefore, determining how DM educational media could have affected them was difficult. Therefore, the study results had external validity, and this might be a generalized intervention for patients with type 2 DM in real-world settings. Nevertheless, our study results were inconsistent with those of Janchai et al. [27], who showed that the participants of the group-based educational program had higher HL levels than the control group who received standard care. In addition, infographics have been a useful teaching tool for patients with DM. Alternatively, they can improve the HL of patients with DM in multiple domains, similar to that of a previous study. Negarandeh reported that both educational strategies (pictorial image and teach back) increased knowledge and adherence to medications and diet among patients with type 2 DM and a low HL (*p* < 0.001) [28]. Chang revealed that the web-based intervention comprised five sets of infographics, and one animation significantly improved the participants’ knowledge (*p* < 0.001), behavioral intention (*p* < 0.001), and self-efficacy (*p* < 0.001) related to substance use prevention [29]. Therefore, further larger-scale studies should be performed to reduce the risk of heterogenicity bias between the two groups. To accurately assess the effect of an education intervention, further research that can develop criteria for patients with low HL levels is expected. In addition, infographics have a more detailed content and a clearer and more attractive design, and more than three infographics should be provided weekly.

However, when evaluated for the effect of educational media through mean differences of the S-TOFHLA and FCCHL scores, this study indicated statistically significant mean differences in scores before and after the intervention. Furthermore, the infographic group had slightly higher scores in mean difference at before and after the provision of educational media, more so than the pamphlet group, although no statistically significant difference in the mean differences in HL scores was found between the two groups. This finding was consistent with those of Negarandeh et al. and Pannak et al. [28, 30], who found statistically significance in the mean differences of HL scores before and after the provision of the education intervention between the intervention group (group-based educational format) and the control group. The current study revealed that the target audience had improving HL levels with the use of online infographics and pamphlets via the LINE application. This is especially beneficial during the COVID-19 pandemic when social distancing was recommended to reduce COVID-19 cases and prevent severe illness in patients with type 2 DM.

Owing to the different of evaluation methods of HL, this study used the Thai version of the S-TOFHLA and FCCHL, which are global questionnaires used to determine HL scores and levels [18, 20]. The S-TOFHLA and FCCHL have both been translated into various languages. The details of both types of tools are as follows: the S-TOFHLA (Thai version) is an HL questionnaire for basic HL assessment that involves numbers and calculations and enables the basic level understanding of type 2 DM at the basic level for adults [19]. The Thai version of FCCHL has tools for assessing comprehensive HL skills, whereas self-assessment of health perceptions and attitudes consists of three categories: functional, communicative, and critical levels [22, 23]. The six skills of HL should be evaluated in each study participant [7]. Therefore, the validity and reliability of the questionnaires, namely, S-TOFHLA and FCCHL, were chosen to provide all six skills of HL assessment.

During this study, both forms of education media were distributed through the LINE application due to the COVID-19 pandemic. Therefore, we could not determine whether the participants had received or read the contents of the infographics or pamphlets. This issue was addressed by contacting all participants via telephone to validate the type of educational media they used. To ensure that the participants had already received and read the educational media, they were contacted weekly to check the status of the sent information via the LINE application. No further information or knowledge was provided during telephone calls. In this study, the distribution of educational media were consistent with that in the study by Mengiste et al. [31], who reported that sources of DM-related information include health professionals, books, internet, brochures (pamphlets), mass media, family, friends, and magazines or newspapers. However, both groups received educational media through the LINE application. Infographics, which are picture-based educational media, might be more appropriate for online distribution than pamphlets. Because infographic functions are visually appealing, brief, and concise, they are easy to understand. Infographics are often shared on social media, rendering them easily accessible by a large group of people [15]. Pamphlets, usually text-based media, are often distributed as printed matter; therefore, the results could differ if they are distributed via an offline platform. hence, further studies regarding the distribution of pamphlets via printed matter are warranted to determine the effect of pamphlets on HL improvement.

### Strengths

This is one of the few studies comparing the effect of online infographics with usual health education on improving HL in patients with type 2 DM. During the COVID-19 pandemic, in which the study was also conducted, infographics and pamphlets were distributed via the LINE application, which was appropriate during the pandemic**.** Moreover, improving HL methods by using the LINE application of infographics or pamphlets is simple and inexpensive and reduces the gathering of people due to the COVID-19 pandemic.

### Limitations and further study suggestions

The current study had a few limitations that could have influenced the study results. First, patients with type 2 DM aged > 60 years were excluded from this study because the authors were concerned about their capability to access the Internet via a mobile device. There are several individuals with DM aged > 60 years. Therefore, further studies should include patients with type 2 DM aged over 60 years. Second, the baseline sociodemographic characteristics of the participants, including age and type 2 DM duration, were heterogeneous. These variables differed significantly between the two groups, possibly due to variations in the randomization phase. Hence, a larger number of patients should be utilized to mitigate this issue. In addition, HL improvement with the LINE application can be observed in patients with type 2 DM who have a smartphone or tablet and those who can use basic and popular applications for Thai adults; however, this may result in a random distribution, with unequal chances for each sample unit of the population because it is only used when an Internet connection is available. Consequently, data were unavailable, while the sample group was not connected to the Internet. In addition, due to time constraints and the COVID-19 situation, clinical outcomes of DM, such as HbA1C and fasting blood sugar (FBS), could not be measured by blood tests. In addition, the final evaluation of the participants in both groups was conducted at their home using an HL questionnaire at 1 week after final educational intervention; therefore, information bias might have arisen because of family members assisting in answering the HL questionnaire. To reduce the chance of this bias, participants were required to answer questions in a private area, without assistance from family members, to accurately represent the participant’s HL outcomes.

Future research should focus on examining the effect of various DM educational programs to enhance HL among patients with type 2 DM. These studies should specifically include participants aged over 60 years. In addition, including a greater number of patients is important to ensure the robustness of the findings. In addition, clinical outcome measurements (i.e., HbA_1_C and FBS) should be performed to measure the effectiveness of DM educational materials. Considering the evolving landscape of healthcare and patient needs, there is a significant opportunity for future studies to explore and develop innovative, evidence-based methods for educating patients with DM, thereby ensuring more effective management and improved outcomes.

## Conclusions

Novel infographics and pamphlets distributed via the LINE application did not significantly differ in achieving adequate HL among patients with type 2 DM who should receive health education about disease control and complication prevention. However, both interventions can enhance and maintain HL levels. Online educational media can be appropriate during the COVID-19 pandemic. Nevertheless, further larger-scale studies should be conducted to examine the impact of other effective DM educational media on HL promotion.

### Supplementary Information

**Additional file 1: Supplementary 1.** Contents of type 2 diabetes mellitus educational media.

**Additional file 2: Supplementary 2.** Three types of educational infographics for type 2 diabetes mellitus.

**Additional file 3: Supplementary 3.** Three types of educational pamphlets for type 2 diabetes mellitus.

## Data Availability

Data are available upon reasonable request. De-identified data collected for this study are available from the corresponding author upon reasonable request.

## Abbreviations

CVI: Content validity index
DM: Diabetes mellitus
FBS: Fasting blood sugar
FCCHL: Functional, Communicative and Critical Health Literacy
HL: Health literacy
IQR: Interquartile ratio
PCU: Primary care unit
SD: Standard deviation
