# Supplementary material for: Effect of online infographics for enhancing health literacy among patients with type 2 diabetes in primary care unit during the COVID-19 pandemic: a randomized controlled trial
Source: BMC Prim Care. 2024 Mar 15;25:87. doi: 10.1186/s12875-024-02335-2 (PMC10941353; doi:10.1186/s12875-024-02335-2)
Supplement: Supplementary file 1 — Additional file 1: Supplementary 1. Contents of type 2 diabetes mellitus educational media. [file 12875_2024_2335_MOESM1_ESM.pdf]

Table 1. Contents of type 2 diabetes mellitus educational media

Diabetic education	Experimental group (infographic)	Control group (pamphlet)
**First domain (week 1)**		
**General knowledge about diabetes**	✓	✓
- Type of diabetes	✓	✓
- Symptoms of diabetes	✓	✓
- Diagnostic criteria	✓	✓
- Goal of control (blood glucose/HbA1C level)	✓	✓
- Risk for diabetes		
**Second domain (week 2)**		
**Complications of diabetes**		
- Acute complications	✓	✓
- Symptoms of hyperglycemia and hypoglycemia	✓	✓
- Early management of hyperglycemia and hypoglycemia	✓	✓
- Chronic complications	✓	✓

**Third domain (week 3)**		
**Self-care behavior in diabetes**		
- Dietary control	✓	✓
- Exercise and physical activity	✓	✓
- Foot care	✓	✓
- Taking medications	✓	✓
