# Supplementary material for: Effect of online infographics for enhancing health literacy among patients with type 2 diabetes in primary care unit during the COVID-19 pandemic: a randomized controlled trial
Source: BMC Prim Care. 2024 Mar 15;25:87. doi: 10.1186/s12875-024-02335-2 (PMC10941353; doi:10.1186/s12875-024-02335-2)
Supplement: Supplementary file 2 — Additional file 2: Supplementary 2. Three types of educational infographics for type 2 diabetes mellitus. [file 12875_2024_2335_MOESM2_ESM.pdf]

# General Knowledge of Diabetes

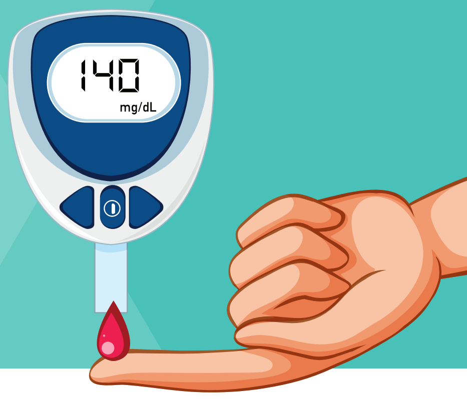


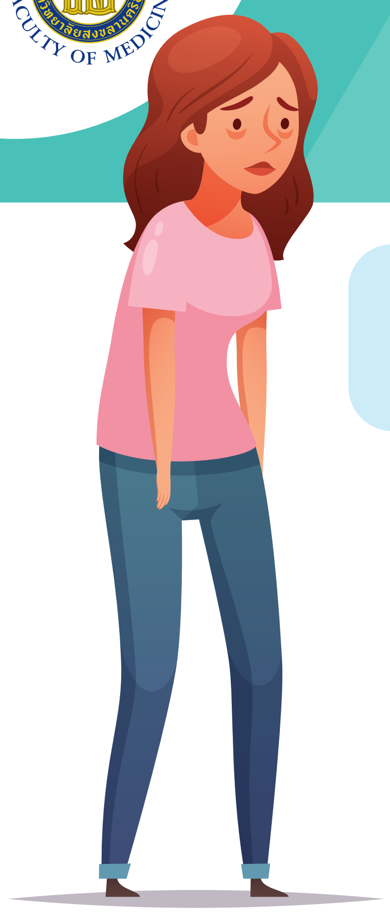

Diabetes is a condition in which blood sugar levels are higher than normal. It is caused by a lack of insulin hormone or decreased effectiveness of insulin due to insulin resistance. Long-term blood sugar elevation will cause complications to various organs; including the eyes, kidneys and nerve

## Types of diabetes

***Type 1*** diabetes (due to autoimmune β-cell destruction, usually leading to absolute insulin deficiency, including latent autoimmune diabetes of adulthood)

***Type 2*** diabetes is present 90-95% of diabetes (due to a non-autoimmune progressive loss of adequate β-cell insulin secretion frequently on the background of insulin resistance and metabolic syndrome)

***Type 3*** Gestational diabetes mellitus (diabetes diagnosed in the second or third trimester of pregnancy that was not clearly overt diabetes prior to gestation)

***Type 4*** Specific types of diabetes due to other causes, e.g., monogenic diabetes syndromes (such as neonatal diabetes and maturity-onset diabetes of the young), diseases of the exocrine pancreas (such as cystic fibrosis and pancreatitis), and drug- or chemical-induced diabetes (such as with glucocorticoid use, in the treatment of HIV/AIDS, or after organ transplantation)

## Symptoms of type 2 diabetes

### Common symptoms

- Severe thirst
- Frequent urination
- Weight loss

### Other common symptoms

- Frequent hunger
- Fatigue
- Slow wound healing
- Tingling pain or loss of sensation in the hands and feet
- Blurred vision
- Frequent infections of the skin, vagina or urinary tract

## How does a doctor diagnose diabetes?

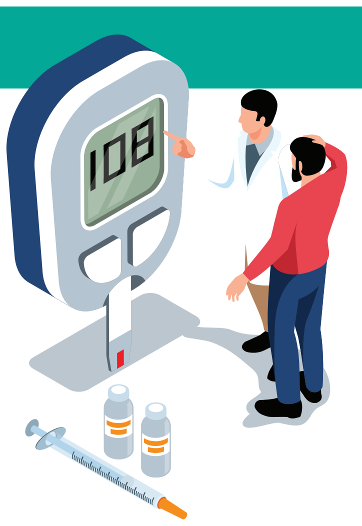


| Laboratory | Normal | Prediabetes | | Diabetes Mellitus |
|---|---|---|---|---|
| | | impaired fasting glucose (IFG) | impaired fasting tolerance (IGT) | |
| Fasting plasma glucose | < 100 mg./dL. | 100-125 mg./dL. | - | > 126 mg./dL. |
| 2 hr-plasma glucose (oral glucose tolerance test) | < 140 mg./dL. | - | 140-199 mg./dL. | > 200 mg./dL. |
| Anytime plasma glucose + clinical symptom of diabetes | - | - | - | > 200 mg./dL. |
| Hemoglobin A1C | < 5.7% | 5.7-6.4% | | ≥6.5% |

## Who is at risk for type 2 diabetes and who should be screened?

- 35+ — Age **≥35 years**
- Lack of **exercise**
- **Obesity** body mass index > 25 kg/m$^2$
- >130 SYS >80 DIA — History of **hypertension**
- HDL cholesterol **< 35 mg/dl**
- **A family history** especially a first-degree relative with type 2 diabetes
- **History of gestational diabetes** or having given birth to a child who weighed >4 kg
- History of **cardiovascular disease**
- Having history of blood sugar levels, that were **between 100 and 125 mg/dL**

Hariri S, Yoon PW, Qureshi N, Valdez R, Scheuner MT, Khoury MJ. Family history of type 2 diabetes: a population-based screening tool for prevention? Genet Med. 2006 Feb;8(2):102-8. doi: 10.1097/01.gim.0000200949.52795.df. PMID: 16481893.

Department of Family and Preventive Medicine
Faculty of Medicine, Prince of Songkla University
Tel. : 074-451330
https://fmpm.medicine.psu.ac.th/

# Complications
## of Type 2 Diabetes Mellitus

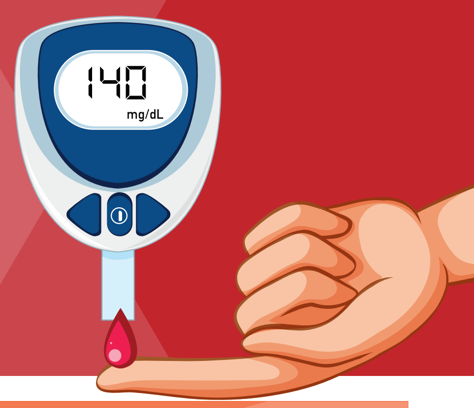


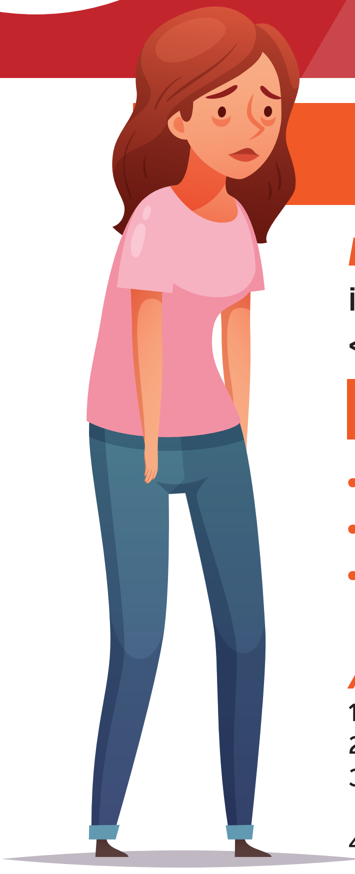

## Acute complications

### Hypoglycemia
is a condition in which the blood glucose level <70 mg/dL.

**Symptoms**

- Palpitation
- Sweating
- Fatigue
- Feeling dizzy
- Confused
- Feeling hungry

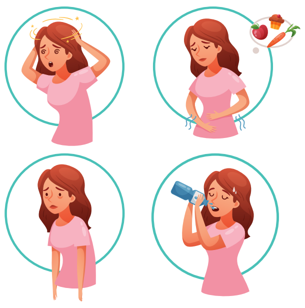

### Hyperglycemia
is a condition in which the blood glucose level >300 mg/dL.

**Symptoms**

Frequent urination, frequent thirst, fatigue, weight loss.

**If the blood sugar level is very high, It can cause the patient to faint or lose consciousness**

***Assessment of patients to determine the risk/stage of complications for consultation/referral***

1. Diabetic nephropathy : UACR ≥300 mg/gC, GFR < 30mL/min/1.73m$^2$
2. Diabetetic retinopathy: severe non-proliferative diabetic retinopathy(NPDR), proliferative diabetic retinopathy(PDR)
3. Diabetic foot ulcer: abnormal foot sensation, decrease peripheral pulses/ABI <0.9 or >1.3,history of foot ulcer(gangrene), previous amputation, intermittent claudication, rest pain
4. Cardiovascular complication: angina pectoris, coronary artery disease, cerebrovascular disease

## What should I do when I have symptoms?

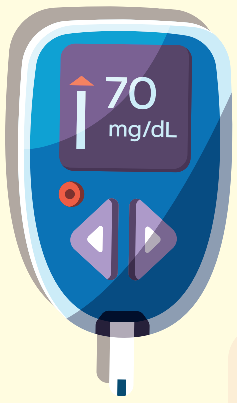


Check with a fingertip glucose meter. If the blood sugar <70 mg/dL, you should do the following:

***In mild cases***

**Drink sweetened beverages**

such as orange juice or soft drinks

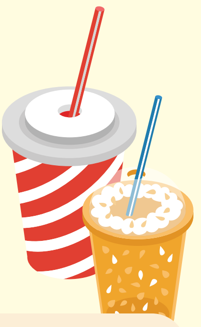

Check your blood sugar level and retake the drink **if symptoms do not improve in 15 minutes.** Additionally, you should visit a doctor if you consistently experience low blood sugar symptoms

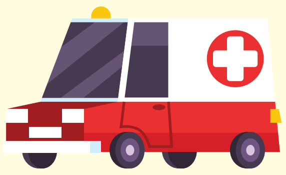

***In case of severe symptoms (unconsciousness)***

**Transport to the nearest hospital as soon as possible**

**Do not give food or drink, because it can cause aspiration**

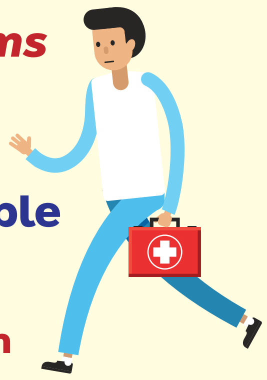

## Chronic complications

**Occurs in diabetic patients, who have had the disease for ≥5 years;**
particularly in those who are unable to control their blood sugar levels

### Complications in small blood vessels

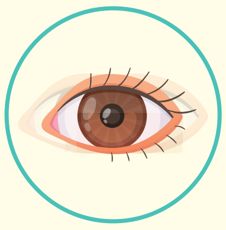

Diabetes
**Retinopathy**

- Blurred vision
- Shadow like vision
- Diplopia

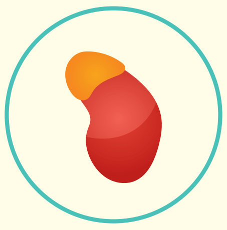

Diabetes
**Nephropathy**

- Initially, the patient is symptom-free, and the urine contains a tiny amount of albumin.
- Foamy urine and edema may also be noticed later

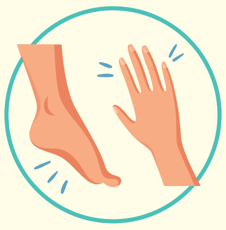

Peripheral
**Neuropathy**

Experience numbness in both hands and feet or a burning sensation. The main microvascular complications of both type 1 and type 2 diabetes mellitus.

### Complications in large blood vessels

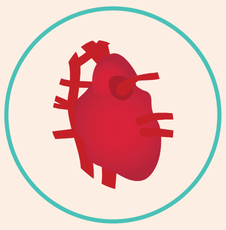

**Coronary heart disease**

Chest pain, Dyspnea on exertion, increased risk of sudden death

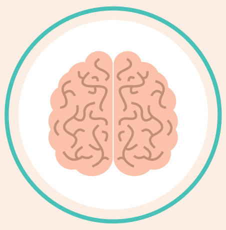

**Stroke**

Weakness in either the upper or lower limbs; or in both, Facial palsy and slurred speech

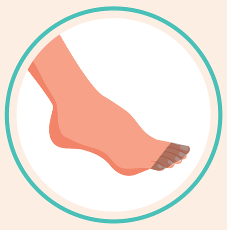

**Peripheral vascular disease**

A risk factor for foot ulcers


Department of Family and Preventive Medicine
Faculty of Medicine, Prince of Songkla University
Tel. : 074-451330
https://fmpm.medicine.psu.ac.th/

# Self-care Behavior for Diabetes

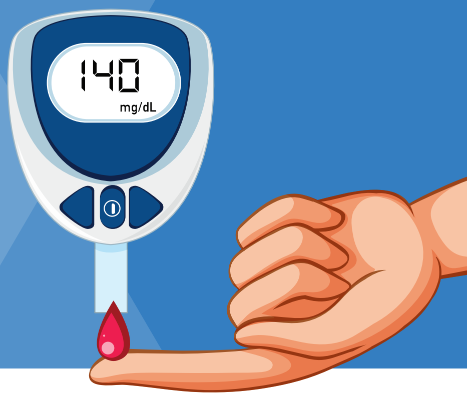


## Dietary control

### Meal preparation: use a simple principle by dividing the plate into portions as follows

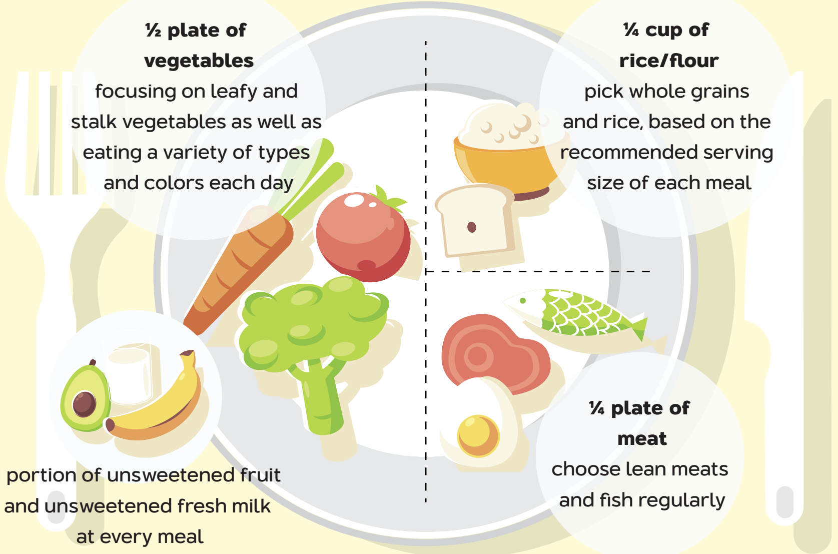

**½ plate of vegetables** focusing on leafy and stalk vegetables as well as eating a variety of types and colors each day

**¼ cup of rice/flour** pick whole grains and rice, based on the recommended serving size of each meal

**¼ plate of meat** choose lean meats and fish regularly

1 portion of unsweetened fruit and unsweetened fresh milk at every meal

### Food that Diabetes can eat without limitation

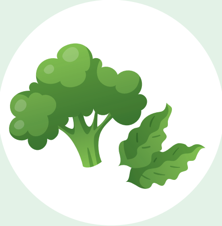

**All kinds of green leafy vegetables**

except starchy root vegetables and pods

### Suitable foods for Diabetes

Although they must be consumed appropriately and limited

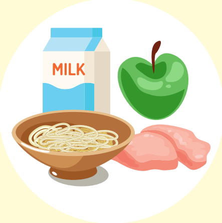


Rice, flour, meat, egg, milk, oil, fruit

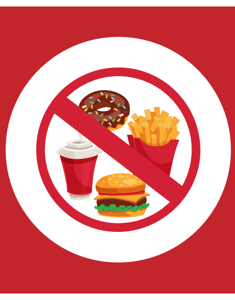

### Foods to avoid

**Sugar, sweets, sweetened beverages, alcoholic beverages, salty foods, fatty meats and fried foods**

## Exercise

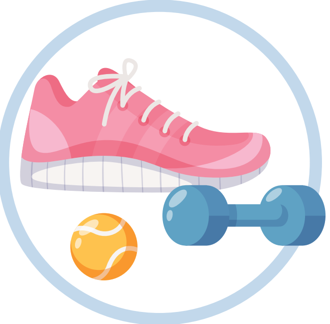

### Proper exercise

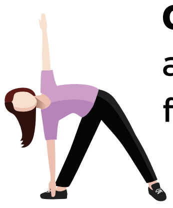

**One should exercise regularly** at least 3-5 times a week for 30-60 minutes.

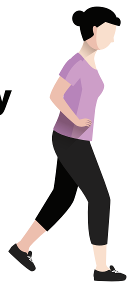

### Caution for exercising in diabetic patients

- Should undergo a general physical before exercise
- Check your feet before and after exercise
- Do not exercise within 1 hour prior to mealtime
- When symptoms start to appear abnormal, stop exercising

## Foot care

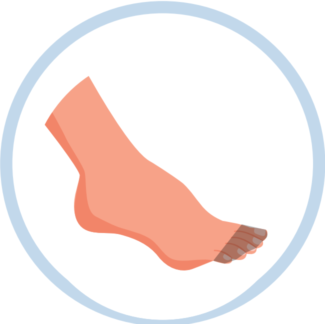

Diabetes causes the blood vessels and nerve endings to deteriorate, which results in foot numbness.
Then there is a chance of easy injury and healing slowly. Because of the reduced blood flow and probable infection,
Foot care guidelines

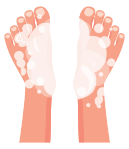

**Wash** your feet at least twice a day

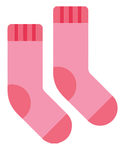

**Wear soft socks** every time you put on your shoes

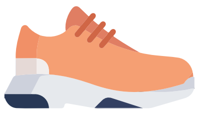

**Choose closed-toe footwear** to reduce impact

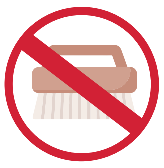

**Do not use a hard-bristled brush** to scrub your feet and nails

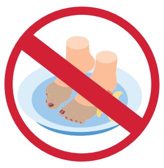

If you wish to soak your feet **do not soak them for too long**

## Taking medicine

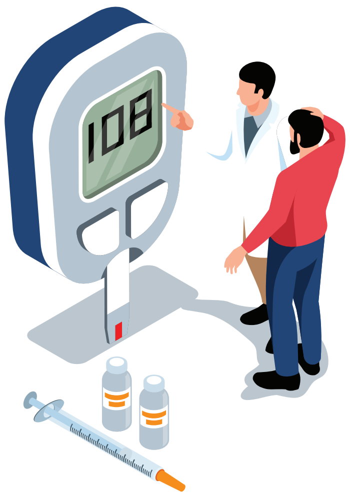


**The medication should also be taken at the same meal every day and routinely.**
**When using diabetes drugs, other concerns to think about include**

- Do not adjust the dosage yourself.
- If there is a delay in eating, there should be milk or food that is easily portable and ready to eat immediately.
- In case of illness and unable to eat normally, you may need milk or fruit juice instead; however, you should not stop taking the medication.

| Name | Type | Dosage(mg/day) | Side effect |
|---|---|---|---|
| Metformin | Biguanides | 500-2550 mg/day | Nausea, Anorexia, Diarrhea |
| Sulfonylurea<br>- Glipizide,<br>- Glimepiride | Sulfonylurea | 2.5-40mg/day<br>1-8mg/day | Hypoglycemia |
| Pioglitazone | Thiazolidinediones | 15-30mg/day | Weight gain, Edema |
| NPH | Insulin | 0.1-0.2 unit/kg/day | Hypoglycemia |

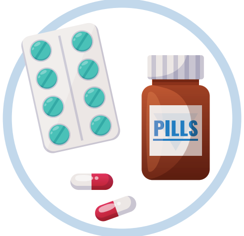



Department of Family and Preventive Medicine
Faculty of Medicine, Prince of Songkla University
Tel. : 074-451330
https://fmpm.medicine.psu.ac.th/
