# Supplementary material for: Effect of online infographics for enhancing health literacy among patients with type 2 diabetes in primary care unit during the COVID-19 pandemic: a randomized controlled trial
Source: BMC Prim Care. 2024 Mar 15;25:87. doi: 10.1186/s12875-024-02335-2 (PMC10941353; doi:10.1186/s12875-024-02335-2)
Supplement: Supplementary file 3 — Additional file 3: Supplementary 3. Three types of educational pamphlets for type 2 diabetes mellitus. [file 12875_2024_2335_MOESM3_ESM.pdf]

Who is at risk for type 2 diabetes and should be screened?

1. Old age: it was found that as people get older, their risk of acquiring diabetes increases. It is suggested that people over 35 in the general population should be screened blood sugar levels to determine risk of diabetes.
2. Lack of exercise: This can increase your risk of type 2 diabetes.
3. Obesity: body mass index greater than 25 kg/m2 or waist circumference > 90 cm in men and > 80 cm in women.
4. There is a family history, especially a first-degree relative with type 2 diabetes.
5. History of gestational diabetes or history of giving birth to a child weighing more than 4 kg.
6. History of hypertension
7. HDL cholesterol less than 35 mg/dl.
8. Had a history of blood sugar level after an 8-hour fasting that were between 100 and 125 mg/dL, which was considered to be pre-diabetes.
9. Have a history of cardiovascular disease, either currently cardiovascular disease.

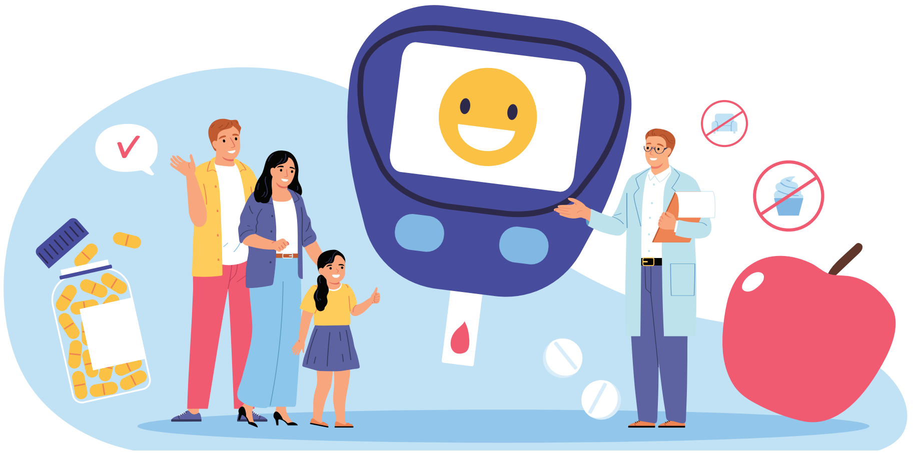

Department of Family and Preventive Medicine

Faculty of Medicine,
Prince of Songkla University

Tel. : 074-451330
https://fmpm.medicine.psu.ac.th/

General Knowledge of Diabetes

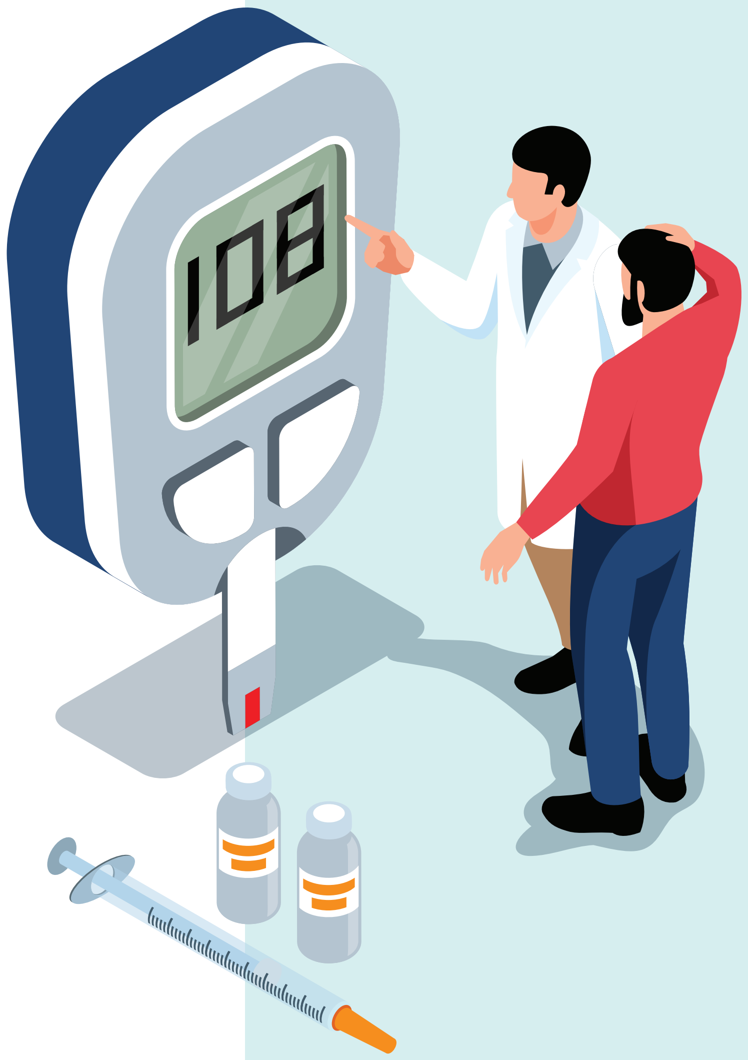

Department of Family and Preventive Medicine
Faculty of Medicine, Prince of Songkla University

General Knowledge of Diabetes

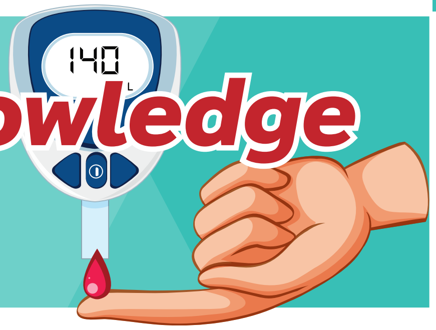

Diabetes is a condition in which blood sugar levels are higher than normal. It is caused by a lack of insulin hormone or decreased effectiveness of insulin due to insulin resistance. Long-term blood sugar elevation will cause complications to various organs, including the eyes, kidneys and nervous system.

Types of diabetes:

Type 1 diabetes (due to autoimmune β-cell destruction, usually leading to absolute insulin deficiency, including latent autoimmune diabetes of adulthood)

Type 2 diabetes is present 90-95% of diabetes (due to a non-autoimmune progressive loss of adequate β-cell insulin secretion frequently on the background of insulin resistance and metabolic syndrome)

Type 3 Gestational diabetes mellitus (diabetes diagnosed in the second or third trimester of pregnancy that was not clearly overt diabetes prior to gestation)

Type 4 Specific types of diabetes due to other causes, e.g., monogenic diabetes syndromes (such as neonatal diabetes and maturity-onset diabetes of the young), diseases of the exocrine pancreas (such as cystic fibrosis and pancreatitis), and drug- or chemical-induced diabetes (such as with glucocorticoid use, in the treatment of HIV/AIDS, or after organ transplantation)

Symptoms of type 2 diabetes

In the early stages, there are no symptoms and can only be diagnosed by blood tests. Severe thirst, frequent urination, and loss of weight despite eating regularly are significant symptoms. Other common symptoms include frequent hunger, fatigue, blurred vision, slow wound healing, tingling pain or loss of sensation in the hands and feet and frequent infections of the skin, vagina or urinary tract.

Who is at risk for diabetes?

The main variables that may contribute to diabetes are increasing age, heredity, obesity, and a lack of exercise, but the specific etiology of the disease is still unknown. There is a higher probability of developing diabetes if a person has more risk factors.

How does a doctor diagnose diabetes?

Diagnosed diabetes, it only be done by blood sugar testing after an 8-hour fasting to determine blood sugar levels. If the blood sugar level is more than 126mg/dL, the test should be repeated. Diabetes is considered if the repeated level stays above 126 mg/dl, but if there are significant symptoms of diabetes mentioned above. Blood test without fasting found that blood sugar higher than 200 mg/dL was considered diabetes.

Diabetes complications

1. Atherosclerosis this results in artery narrowing or blockage and causes ischemia in various organs, which leading to diseases such ischemic heart disease and cerebrovascular disease.

2. Deteriorating kidneys: for diabetics, the change is significant. This can result in renal failure.

3. Macular Degeneration: may cause vision loss

4. Peripheral nerve degeneration that results in loss of sexual feeling and numbness at the tips of the hands and feet

From the complications that mentioned above, all are serious complications or degenerative conditions of various tissues due to a long-term disease. Therefore, normal blood sugar control is a way to prevent complications.

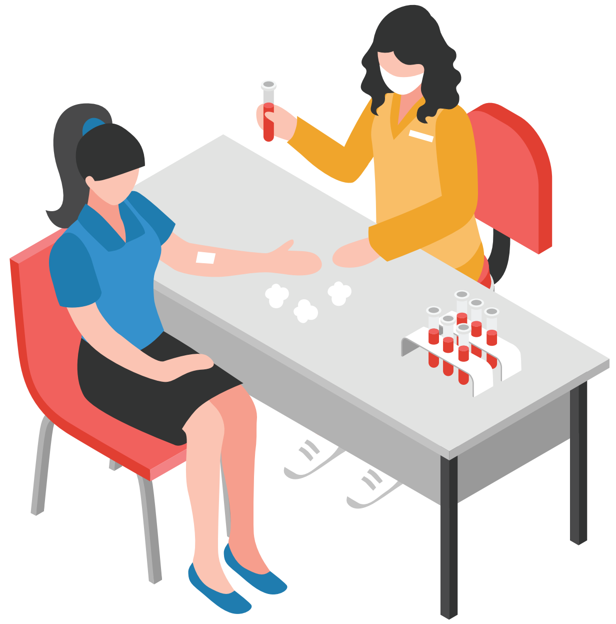

• ***Diabetes nephropathy:***

It is a major cause of illness and death in both type 1 and type 2 diabetes mellitus. Incidence and progression of renal complications. It also associated with blood glucose control and blood pressure control.

Sign and Symptom

- Initially, the patient is symptom-free. The urine contains a tiny amount of albumin, It was found in the urine on a daily basis in amounts of 30-300 mg.
- Foamy urine and edema may also be noticed later, when the amount of protein that leaks out increases. At this stage, high blood pressurevalso detected. (the amount of albumin in the urine at this stage is greater than 300 mg per day)
- Following that, if it is not appropriately treated, kidney function will decline, eventually leading to chronic renal failure. until the need for hemodialysis or dialysis treatment

• ***Diabetes neuropathy:***

The patient will have toe numbness. It is a risk factor for foot ulcers in diabetics. especially patients with peripheral vascular disease. Patients may end up with their fingers or a portion of their legs amputated as a result. which factor will lead to future disability. Blood sugar, high blood pressure, smoking, and other risk factors are associated with nerve problems.

Sign and Symptom

In the early stages, the patient may experience numbness in both hands and feet, some people will have a burning pain or sharp pain. The symptoms mostly manifest at night. Later on, the pain subsides but there will be numbness and decreased sensation. In addition, some people may experience weakness.

Department of Family and Preventive Medicine

Faculty of Medicine,
Prince of Songkla University

Tel. : 074-451330
https://fmpm.medicine.psu.ac.th/

Acute Complications of Diabetes

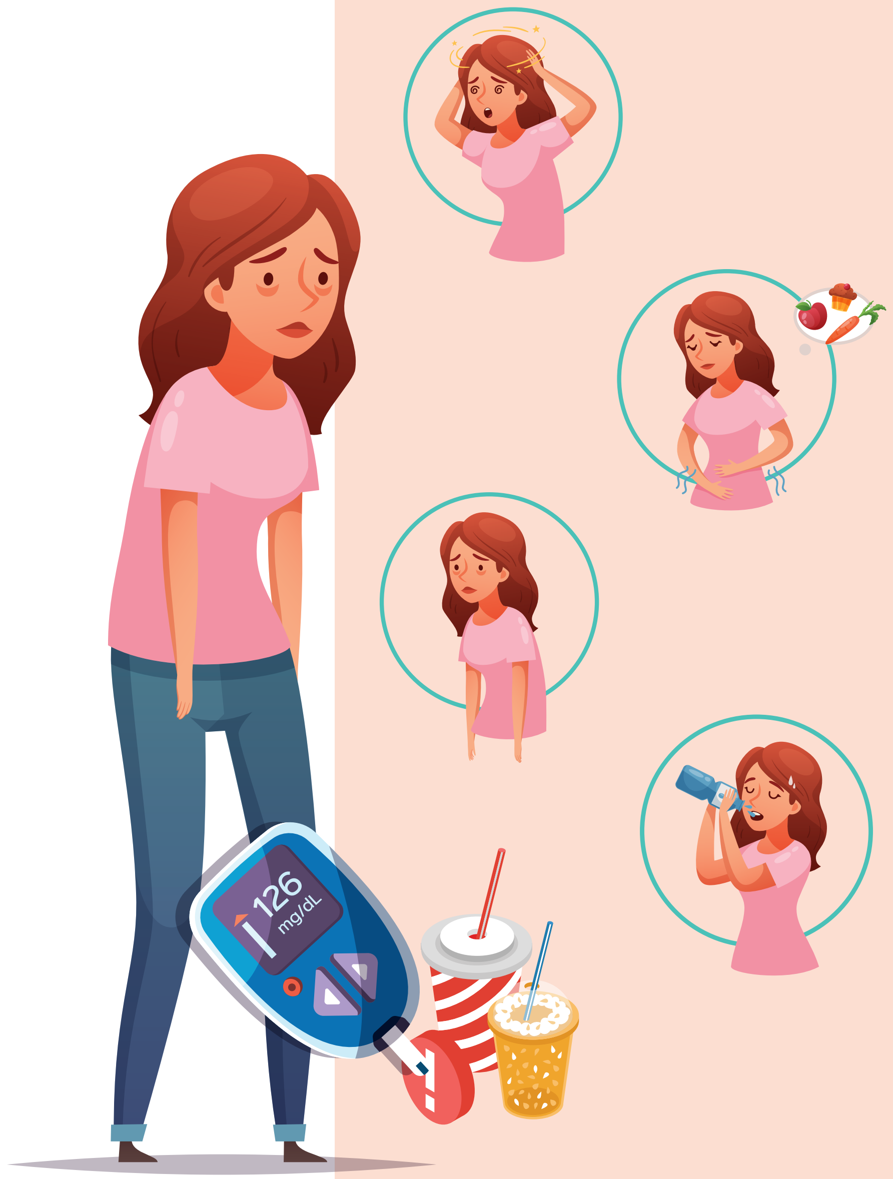

Department of Family and Preventive Medicine
Faculty of Medicine, Prince of Songkla University

Acute Complications of Diabetes

Hypoglycemia

Hypoglycemia in diabetic patients is a condition in which the blood glucose level less than 70 mg/dL.

• Symptom

• Palpitation • Feeling Dizzy • Sweating
• Confused • Fatigue

Hyperglycemia in diabetic patients is a condition in which the blood glucose level is greater than 300 mg/dL.

• Symptom

frequent urination, frequent thirst, fatigue, weight loss. If the blood sugar level is very high, it can cause the patient to faint or lose consciousness.

• How should I do when I have symptoms?

check with a fingertip glucose meter. If the blood sugar is less than 70 mg/dL You should do the following.

• In mild cases:

drink sweetened beverages such as orange juice, soft drinks, check your blood sugar level and retake the drink ***if the symptoms don't improve in 15 minutes. However, you should visit a doctor if you consistently experience low blood sugar symptoms.***

• In case of severe symptoms (unconsciousness):

Take the patient to the nearest hospital as soon as possible, do not give some food or drink to the unconscious person because it can cause aspiration.

Chronic Complications of Diabetes

Occurs in diabetic patients who have had the disease for at least five years or more, particularly in those who are unable to control their blood sugar levels.
The complications are divided into

1. Complications in small blood vessels include:

- Diabetes retinopathy
- Diabetes nephropathy
- Diabetes neuropathy

2. Complications in large blood vessels include:

- Coronary heart disease
- Stroke
- Peripheral vascular disease , which increases the risk of diabetic foot ulcers.

• Coronary heart disease:

The patient could feel chest pain, dyspnea on exertion. Symptoms of a heart attack can increase risk of sudden death.

• Stroke

Weakness in either the upper or lower limbs or in both. The symptoms are frequently sudden, include difficulties on movement , facial palsy, slurred speech, etc.

• Diabetes retinopathy:

Diabetic patients who cannot control their sugar levels to be within the appropriate limits will cause complications to the retina and increase the risk of blindness.

Sign and Symptom

- Blurred vision caused by abnormal refraction of the lens while blood sugar is high or caused by cataracts or caused by retinal changes, which condition without proper treatment will eventually lead to blindness in the patient
- see shadows obscuring the image, which is caused by bleeding in the vitreous of the eye.

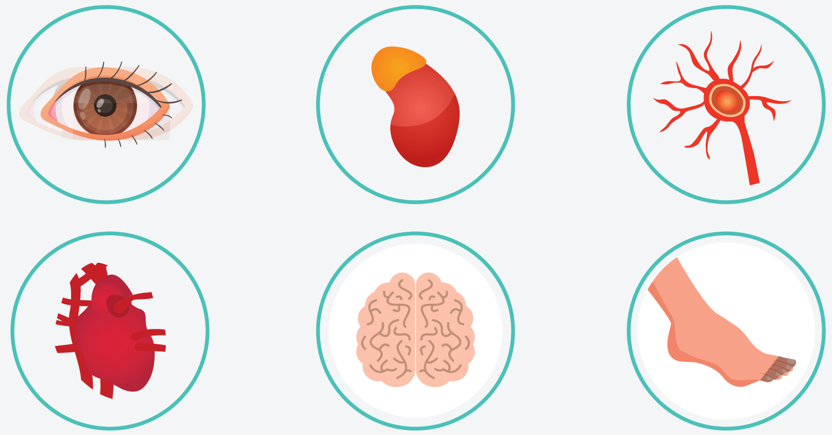

Foot care

Diabetes causes the blood vessels and nerve endings to deteriorate, which results in foot numbness. If stepping on sharp objects or touching hot things does not feel, then there is a chance to easily injure and heal slowly. Because of the reduced blood flow and probable infection, therefore diabetic patients need special foot care.

Foot care guidelines

- Wash your feet at least twice a day.
- Do not use a hard-bristled brush to scrub your feet and nails.
- If you wish to soak your feet, don't soak them for too long, and use room temperature water instead of heated water, may blister easily
- Wipe your feet clean and dry, paying special attention to the finger-web that needs to stay dry.
- Your fingernails should be trimmed to match your fingertips. If there is inflammation, you need to see a doctor immediately.
- Wear soft socks every time you put on your shoes, to prevent the occurrence of wounds and socks should be changed every day.
- Choose closed-toe footwear to reduce impact.
- To avoid shoe bites after purchasing a new pair of shoes, wear them for no longer than 2 hours before switching to the old pair during the first 2 weeks.

Using medication

The medication should also be taken at the same meal every day and routinely. When using diabetes drugs, other concerns to think about include:

- Do not adjust the dosage yourself, should be done by a physician
- If there is a delay in eating. There should be milk or food that is easily portable and ready to eat immediately.
- In case of illness, unable to eat normally, you may need milk or fruit juice instead, should not stop taking the medication.
- In case of diabetes and pregnancy should see a doctor immediately to temporarily adjust oral diabetes medications to insulin injections After giving birth and breastfeeding is stopped, oral tablets can be given again.

Department of Family and Preventive Medicine

Faculty of Medicine,
Prince of Songkla University

Tel. : 074-451330
https://fmpm.medicine.psu.ac.th/

Self-care Behavior for diabetes

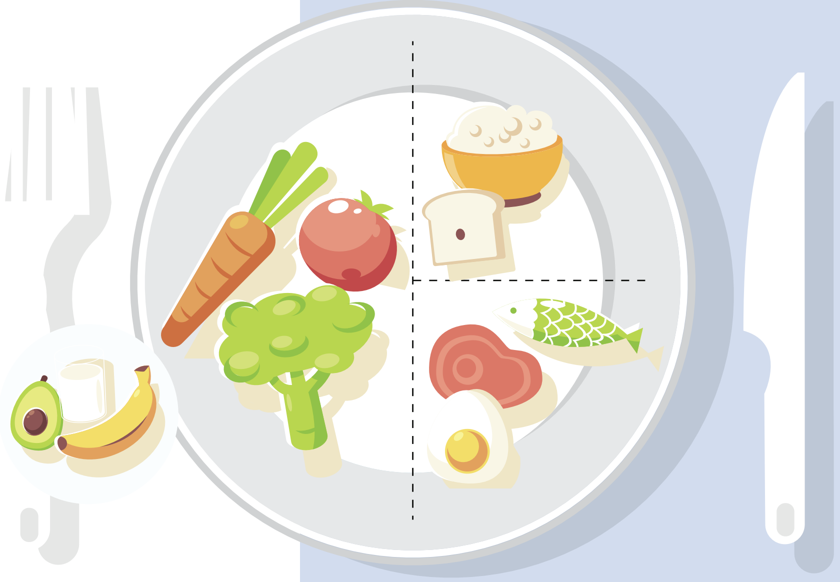

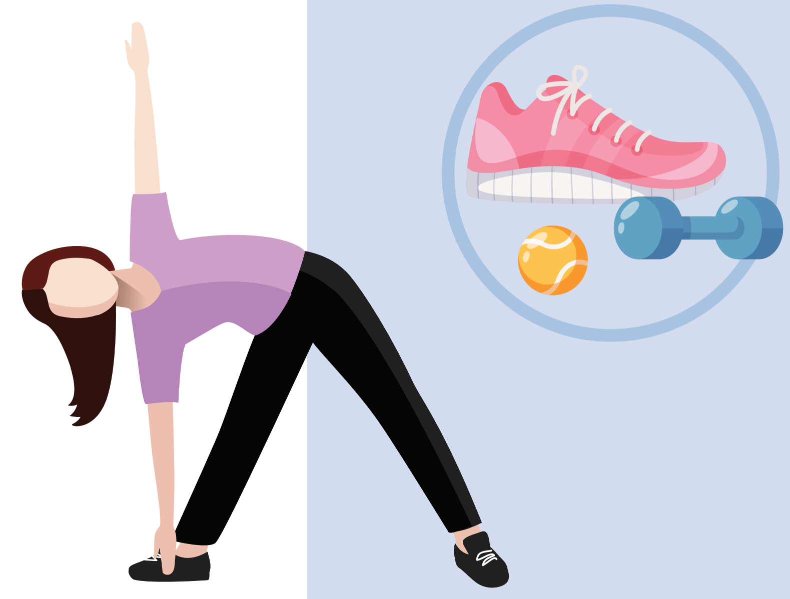

Department of Family and Preventive Medicine
Faculty of Medicine, Prince of Songkla University

Self-care Behavior
for diabetes

Dietary control

Diet is the key to diabetes control, and a regular diet control can help in the following

- body weight is within the acceptable range
- Maintain a normal range for blood sugar
- prevent any complications
- a long and healthy life

Suitable foods for diabetics but must be consumed appropriately and limited

Flour : rice, sticky rice, noodles, bread, macaroni, taro, corn, potato, vermicelli.
Meat : choose lean meat, cooking should be cooked by boiling, steaming or grilling.
Eggs : can eat 2-3 eggs per week, egg yolks should be avoided if your cholesterol is excessive, but you can eat egg whites every day.
Milk : Choose low-fat milk to drink.
Avoid using animal-based oils : fried foods and also foods with coconut milk as an ingredient.
Fruits : avoid very sweet fruits such as oranges, bananas, papayas, watermelons, rambutans, pineapples, avoid preserved and canned fruits.

Food that diabetics can eat without limitation

Vegetables : all kinds of green leafy vegetables can be eaten without limits, such as lettuce, gourd, zucchini, bok choy, basil leaves, mint leaves.
Except (starchy root vegetables and pods) such as pumpkin, banana flower, yard long bean, pea, baby corn, bell pepper, winged bean, onions, carrots, water chestnuts, baby coconut shoots, bamboo shoots

Foods to avoid

- Avoid all kinds of sugar such as granulated sugar, palm sugar, honey or fruit juice.
- All kinds of desserts such as cakes, cookies, Thai desserts.
- Sweetened beverages such as sweetened milk, flavored milk, soft drinks, soft drinks, fresh sugar
- Avoid drinking alcoholic beverages.
- Avoid salty foods and instant foods such as sausages and instant noodles.
- Avoid fatty foods including offal, pork belly, egg yolk, chicken skin, duck skin, and pork knuckle.
- Avoid fried food

Meal preparation for each meal : use a simple principle by dividing the plate into portions as follows:

- **½ plate of vegetables: focusing on leafy and stalks vegetables, eat a variety of types and colors each day.**
- **¼ cup of rice/flour: pick whole grains and rice based on the recommended serving size each meal.**
- **¼ plate of meat: choose lean meats and fish regularly.**
- **1 portion of unsweetened fruit and unsweetened fresh milk every meal.**

Exercise

Proper exercise

- Should exercise regularly at least 3-5 times a week for 30-60 minutes.
- You should do aerobic exercise. It involves continuous limb movement without a lot of impact, such walking, cycling, Tai chi, swimming or water walking.
- An easy way to notice that the exercise is not too heavy is while exercising still able to talk to others. Stop immediately if you feel dizzy or have chest pain.
- Should exercise in an area with good ventilation. neither too cold nor too hot.
- Every time you exercise, check your feet.

Caution for exercising in diabetic patients

- should get a general physical before exercise.
- begin with less exercise and then gradually increase.
- Check your feet both before and after exercise, should check for any sores, abrasions, bruising, swelling, or redness.
- Put on relaxed, comfy shoes with soft soles.
- Dress comfortably and loosely.
- Do not exercise within 1 hour before meal time.
- As soon as symptoms start to appear abnormal, stop exercising.
